# A preliminary clinical trial using flowable glass-ionomer cement as a liner in proximal-ART restorations: The operator effect

**DOI:** 10.4317/medoral.18497

**Published:** 2013-03-25

**Authors:** Clarissa C. Bonifácio, Daniela Hesse, Marcelo Bönecker, Cor Van Loveren, W E. Van Amerongen, Daniela P. Raggio

**Affiliations:** 1 Department of Conservative and Preventive Dentistry, Academic Centre for Dentistry Amsterdam (ACTA), Amsterdam, The Netherlands; 2 Department of Pediatric Dentistry, School of Dentistry, University of São Paulo, São Paulo, Brazil

## Abstract

Objectives: This in vivo study was carried out to assess the influence of the operator experience on the survival rate of proximal-ART restorations using a two-layer technique to insert the glass-ionomer cement (GIC). Study Design: Forty five proximal cavities in primary molars were restored in a school setting according to the ART technique. The cavities were restored by two operators with Ketac Molar Easymix, and received a flowable layer of GIC prior to a second GIC layer with a regular consistency. The operators had different clinical experiences with ART (no experience or two years of experience), but both completed a one-week training to perform the restorations and the GIC mixing in this study. Results: After a 12-month follow-up, 74% of the restorations survived; the main reason for failure was bulk fracture or total loss of the restoration.There was no operator influence (log-rank test p=0.2) Conclusion: The results encourage future well designed controlled clinical trials using the two-layer technique for insertion of GIC in proximal-ART restorations, after training the operators.

** Key words:**Atraumatic Restorative Treatment (ART), Glass-ionomer, proximal restorations.

## Introduction

Atraumatic Restorative Treatment (ART) is an alternative approach to manage dental caries. Studies show good performance for single surface restorations made with high-viscosity glass-ionomer cements (GIC) and the ART approach (1-3). However, the performance of proximal-ART restorations is still far from ideal (4-6). An important factor that may contribute to the failure rate of proximal-ART restoration is the highly viscous consistency of the GIC, which makes it a cement with complex handling and insertion characteristics (7). These characteristics can lead to an incorrect adaptation to the tooth surface, resulting in cervical gaps and loss of the restoration (8-11). Recent laboratory studies showed that insertion of a thin flowable GIC layer within proximal cavities prior to the insertion of a regular high-viscosity GIC layer (two-layer technique), can improve the material’s adaptation to tooth structures and increase the bond strength to sound dentin (12,13).

The success of ART-restorations can be influenced by many causative factors; the most often reported is an operator effect (1,14-18). The influence of the operator includes the proper use of hand instruments, cavity conditioning, manipulation of the restorative material and, in cases of multi-surface restorations, factors such as correct matrix band application and sufficient cavomaterial adaptation (18). Differences in individual skills are always expected (17) and it is likely that inexperienced or inadequately trained operators would perform worse than well trained ones (19).

Inserting the GIC in two layers with two different consistencies may enhance the operator/assistant effect for proximal-ART restorations, and it is not known whether this two-layer technique would be applicable to a school setting without facilities like proper illumination, suction, and dental chair. In this study, we proposed to use this new technique for insertion of GIC in proximal cavities, and aimed to assess the influence of operator effect in the survival rate of proximal-ART restorations using the two-layer technique in primary molars. The null hypothesis tested was that there is no difference in the survival rate of two-layered proximal-ART restorations made by two operators who had different clinical experience with ART (no experience or two years of experience), both of them having followed the same training.

## Material and Methods

After examining 232 children participating in an ART class II (proximal cavities) study in the city of Itatiba (State of São Paulo, Brazil), we selected ones with an ART-restoration that had failed (restoration not present) within the first six months after placement. The selected occlusal-proximal cavities were in primary molars. Exclusion criteria were non-cooperative behaviour, pulp exposure, history of pain, presence of swelling or fistula, and mobility of the tooth. Forty five five-to-eight year old children were selected. Written consent was obtained from the parents, and this study was approved by the local Research Ethical Committee.

The operators were one dentist who had two years experience with ART, and one final year dental student who had no previous experience with ART. They both received the same training to perform ART and mix the GIC according to the ART protocol and also to the specific technique used in this study. The training consisted of theoretical lectures (12 hours), clinical demonstrations (4 hours), supervised practice in extracted primary molars (4 hours), and supervised practice in the school enviroment (20 hours). Both operators were assisted by final year dental students who attended to the same training course. The patients were allocated randomly to one of the operators, and they were all enrolled in an oral-health program.

No local anaesthesia was used. Infected carious tissue was removed with hand instruments, and the cavities were restored with Ketac Molar Easymix (3M/ESPE, Seefeld, Germany) using a metallic matrix band and a wedge. The cavity-dimensions were measured using the graduations on the Michigan’s O with Williams marks periodontal probe (20). All cavities received a pre-treatment with diluted Ketac Molar™ Easymix liquid (10 s). A first layer of GIC with flowable consistency (powder/liquid ratio 1:2) was applied. The second layer was mixed according to the manufacturer’s instructions (powder/liquid ratio 1:1) and inserted in the cavity before the final setting of the first layer (12). After the press-finger technique, the excess of material was removed. The restorations were evaluated after one, six, and twelve months according to the ART criteria adapted for proximal restorations (9). All evaluations were performed by one independent evaluator, trained and calibrated by a benchmark (Kappa = 0.89).

Statistical analysis were carried out using Stata 11.2 software (StataCorp, Texas, USA). The results were tested using linear-regression analysis, Kaplan-Meier survival, and a log-rank test at a 95% confidence level.

## Results

At the 12 month follow-up, the survival rate was 74% and the lost to follow-up was 13%.  Table 1 shows the survival/ failure percentages at six- and twelve-month follow-ups.

**Table 1:**
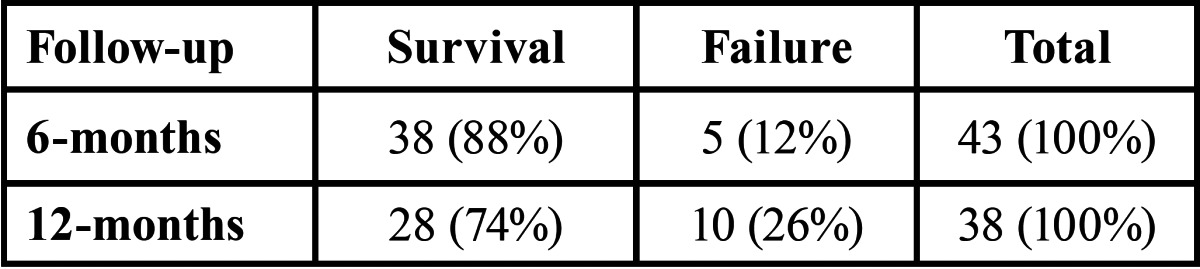
Survival rate and failure of proximal restorations performed with a two-layer technique, after six and twelve months.

Operator 1 performed 21 (47%) of the restorations while operator 2 performed 24 (53%). Of the 45 total restorations, 20 (44%) were placed in the lower jaw and 25 (56%) in the upper jaw; 20 (44%) were on the left side and 25 (56%) on the right side of the mouth; 33 (73%) involved the distal surface and 12 (27%) involved the mesial surface of the element. After one year, 38 restorations were evaluated; of these, 17 (45%) were from operator 1 and 21 (55%) were from operator 2; 18 (47%) were in the lower jaw and 20 (57%) in the upper jaw; 17 (45%) were on the left side and 21 (55%) on the right side of the mouth; 29 (76%) involved the distal surface and 9 (24%) the mesial surface of the element. Linear regression analysis showed no influence of any of these variables on the survival rate of the restorations.

**Figure 1:**
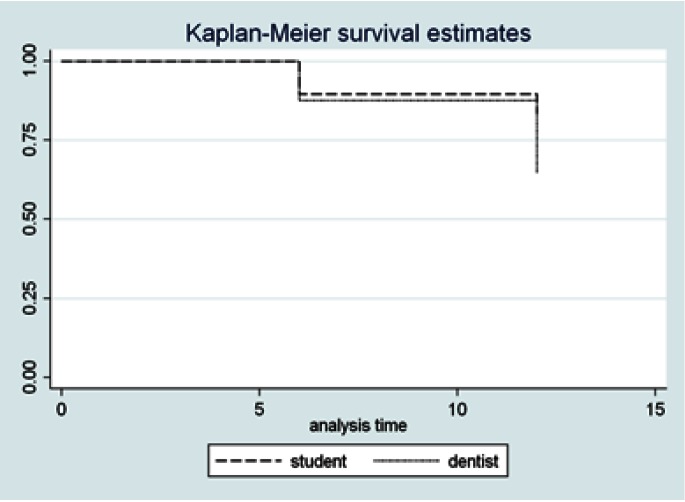
Estimated cumulative survival of the two-layer proximal-ART restorations per operator.

The estimated cumulative survival per operator is presented in figure 1. The log-rank test confirmed the absence of operator influence on the restoration survival rate (p = 0.2). Therefore, we failed to reject the null hypothesis tested.

## ....................

This study investigated whether the insertion of a fluid GIC layer within proximal cavities prior to insertion of a regular GIC layer would be applicable to a school setting, and if this new technique, in this specific setting, would have an operator effect. The results showed an acceptable survival rate, and no operator effect over the time period investigated (12 months). The dropout rate was similar to the ones reported in the literature (21,22). Nevertheless, we acknowledge the limits of our study as having a small sample size, short evaluation period, and the lack of a control group for comparison. The absence of a control group would be a problem if the aim was to compare the retention rate of this restoration against restorations made with the currently used insertion GIC insertion technique in proximal-ART restorations. Yet, in this study, we first aimed to evaluate if we could apply this technique in a field clinical study, using non-experienced operators, so we focused on the survival rate of the restorations per operator. A survival rate of 74% at the first year suggests that this technique should be further investigated. The fact that the cavities had already been previously restored may influence the results, as all the cavities had a second chance to be cleaned, and had volumes between 8 and 16 mm3 after preparation, which are the cavity sizes thought to have the best chance of survival for proximal-ART restorations (23).

An operator effect on the survival of proximal-ART restorations has been previously reported, associating experience with higher survival rates (16-18). We expected that the more sensitive the insertion technique using flowable GIC as a liner, the greater the influence of the operator would be; but this was not the case, as no significant difference was found between the survival rates for the two operators. This lack of difference may be attributed to the fact that both operators and assistants followed a comprehensive training course. This finding suggests that the two-layer technique may have no additional effect on the operator regarding the failure of proximal-ART restorations.

High viscous GIC are difficult to handle and can lead to inadequate adaptation to the cavity walls and cervical gaps (13), both of which contribute to restoration failure (9,10). To improve the GIC adaptation and reduce secondary caries occurence, a flowable layer prior to the insertion of a conventional layer is being proposed in the present study.

The main reason for failure was bulk fracture or total loss of the restoration ( Table 2), which is in accordance with previous literature reports (17,21,24,25). Bulk fractures are generally related to the mechanical properties of the GIC; the use of a flowable layer as a liner might contribute to reduce this property, as the final mixture lead to fewer glass particles. However, Fonseca et al. (26) reported no differences in the diametral tensile strength of conventional GIC when the powder/liquid ratio was reduced by 50%.A disadvantage of the two layer technique might be that the second layer will not adhere to the first layer, contributing to bulk fractures. There are also patient related factors which may influence the survival of proximal-ART restorations, such as cooperative behavior and saliva flow. Moreover, post-restoration meal consumption of a “hard consistency” may influence on the survival rate of proximal-ART restorations (27). The patients were instructed not to eat for one hour after the restoration was placed; however, it was not possible to supervise them. Given the age of the patients, it is, therefore, possible that our instructions were not strictly followed.

**Table 2:**
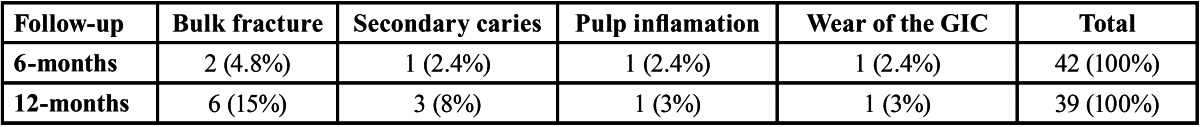
Main reasons for restoration failure in each period.

The literature shows that the 12-month survival rate of proximal-ART restorations in primary posterior teeth ranges between 12% and 88%, for studies conducted in schools (5,17,28,29). Our results showed a survival rate of 74% after one year (Fig. 1). To con-firm the potential improvements delivered by the two-layer technique of applying GIC in ART proximal cavities, further studies, i.e. controlled clinical trials and investigations of the mechanical and adhesive properties of this two-layered GIC, should be conducted.

Based on the results that there was no difference in the survival rate of two-layered proximal-ART restorations made by two operator who had different clinical experience with ART we can conclude that this technique can be applied in a school setting by trained operators.
